# The characteristics of *mcr*-bearing plasmids in clinical *Salmonella enterica* in Sichuan, China, 2014 to 2017

**DOI:** 10.3389/fcimb.2023.1240580

**Published:** 2023-08-29

**Authors:** Xinran Sun, Lin Zhang, Jiantong Meng, Kai Peng, Weifeng Huang, Gaopeng Lei, Zhiqiang Wang, Ruichao Li, Xiaorong Yang

**Affiliations:** ^1^ Jiangsu Co-Innovation Center for Prevention and Control of Important Animal Infectious Diseases and Zoonoses, College of Veterinary Medicine, Yangzhou University, Yangzhou, China; ^2^ Institute of Comparative Medicine, Yangzhou University, Yangzhou, China; ^3^ Center for Disease Control and Prevention of Sichuan Province, Chengdu, Sichuan, China; ^4^ Center for Disease Control and Prevention of Chengdu City, Chengdu, China

**Keywords:** *Salmonella* enterica, colistin resistance, *mcr*, plasmid, genomics

## Abstract

*Salmonella* is one of the most important zoonotic pathogens and a major cause of foodborne illnesses, posing a serious global public health hazard. The emergence of plasmid-mediated *mcr* genes in *Salmonella* has greatly reduced the clinical choice of salmonellosis treatment. The aim of this study was to investigate the plasmid characteristics of *mcr*-positive Salmonella identified from patients in Sichuan, China during 2014 to 2017 by whole genomes sequencing. In this study, a total of 12 *mcr*-positive isolates (1.15%, ; *mcr*-1, n=10; *mcr*-3, n=2) were identified from 1046 Salmonella isolates using PCR. Further characterization of these isolates was performed through antimicrobial susceptibility testing, conjugation assays, whole genome sequencing, and bioinformatics analysis. The *mcr-1* gene in these isolates were carried by three types of typical *mcr-1*-bearing plasmids widely distributed in Enterobacteriaceae (IncX4, IncI2 and IncHI2). Of note, two mcr-1-harboring IncHI2 plasmids were integrated into chromosomes by insertion sequences. Two *mcr-3*-bearing plasmids were IncC and IncFIB broad-host-range plasmids respectively. Genetic context analysis found that *mcr-1* was mainly located in Tn*6330* or truncated Tn*6300*, and *mcr-3* shared a common genetic structure *tnpA-mcr-3-dgkA-ISKpn40*. Overall, we found that *mcr* gene in clinical *Salmonella* were commonly carried by broad-host plasmids and have potential to transfer into other bacteria by these plasmids. Continuous surveillance of MDR *Salmonella* in humans and investigation the underlying transmission mechanisms of ARGs are vital to curb the current severe AMR concern.

## 1 Introduction

Colistin is a polypeptide antimicrobial with a narrow antibacterial spectrum that is largely effective against Gram-negative bacteria and has long been used in clinical medicine and animal husbandry (Landman et al., 2008; Biswas et al., 2012). In livestock industry, it was allowed and widely used for non-therapeutic usage, such as growth promotion, in many countries (Li et al., 2006). In clinical practice, it was commonly used as a non-preferred treatment for multidrug-resistant (MDR) Gram-negative bacteria due to its toxicity to human previously (Conway et al., 1997). However, the emergence of carbapenem-resistant Enterobacteriaceae (CRE) had limited the clinical treatment options. The World Health Organization (WHO) listed colistin as the highest priority critically important antibiotic fighting against CRE. The emergence of plasmid-medicated colistin resistance gene, *mcr-1*, in animal *Escherichia coli* in 2015 fundamentally changed the administration practice of colistin (Liu et al., 2016). Several countries have banned the use of colistin as an animal growth promoter (Wang et al., 2020). However, the current global prevalence of the mobile colistin resistance gene *mcr-1* and its variants are still a serious concern (Wang et al., 2017; Sun et al., 2018). To date, ten different *mcr* variants (*mcr-1* to *mcr-10*) have been identified in different bacteria isolated from animals, foods, humans, and the environment (Hussein et al., 2021). The majority of them were found in plasmids, suggesting that *mcr* variants could be transmitted across intraspecific and interspecific bacteria. The *mcr-1* gene was shown to be more prevalent in bacteria from animal sources than that from patients with nosocomial illnesses (Huang et al., 2017; Tian et al., 2017). This phenomenon may be related to the different exposure of colistin in different settings.

*Salmonella* is a group of gram-negative bacteria found in the intestinal tract of humans and animals as well as in the environment that can cause gastrointestinal diseases and fever called salmonellosis (Lin et al., 1995; Holschbach and Peek, 2018). Recently, a global systematic meta-analysis estimated that approximately 15% of patients with non-typhoidal *Salmonella* invasive disease die (Marchello et al., 2022). In some developing countries, infections caused by *Salmonella* are endemic and represent a serious public health hazard (Abd El-Ghany, 2020). Most healthy people infected by *Salmonella* can recover without specific treatment and do not require treatments of antimicrobials. However, young children and people with weakened immune systems may have an increased risk of developing bacteremia, meningitis and osteomyelitis (Marchello et al., 2022). Although *mcr* genes have seriously spread in Enterobacteriaceae, colistin resistance *Salmonella* were significantly less than *E. coli* and *Klebsiella pneumoniae*. Nonetheless, colistin resistance has emerged in clinical *Salmonella* in many countries, and *mcr-1* and *mcr-3* were the most common variants (Portes et al., 2022). The public health risk caused by colistin-resistant *Salmonella* has increasingly arisen. However, there are few systematic investigations of the plasmid characteristics in *mcr*-positive clinical *Salmonella* (Shen et al., 2021; Portes et al., 2022). In this study, we comprehensively investigated the characteristics of *mcr*-bearing plasmids in clinical *Salmonella* isolates isolated from Sichuan province during 2014 to 2017.

## 2 Materials and methods

### 2.1 Bacterial isolates

The clinical *Salmonella* isolates were collected from different hospitals in Sichuan province, China between 2014 and 2017. The *Salmonella* isolates were recovered from patients following the previous methods (Xia et al., 2009). The *mcr*-positive isolates were screened by the multiplex PCR method and confirmed by Sanger sequencing (Rebelo et al., 2018). All *mcr*-positive isolates were stored at -80°C in LB broth containing 20% glycerol for further investigation.

### 2.2 Antimicrobial susceptibility testing and conjugation assay

The MICs (minimum inhibitory concentrations) of *mcr*-positive isolates against different antimicrobials, including kanamycin, ciprofloxacin, meropenem, ampicillin, colistin, enrofloxacin, tetracycline and ceftiofur, were tested using broth microdilution as per Clinical and Laboratory Standards Institute (CLSI) standards (CLSI, 2020). *E. coli* ATCC25922 was used for quality control. In order to test the transferability of *mcr* genes, conjugation experiments were performed using *E. coli* C600 (Rif^R^) as recipients. Briefly, the donor and recipient strains were grown in LB broth until they reached the logarithmic growth phase, then mixed at the ratio of 1:1 and cultured overnight on LB agar plates. The transconjugants were screened on LB agar plates with rifampin (300 mg/L) and colistin (2 mg/L), and validated using PCR targeting the *mcr* genes and 16S rDNA gene sequencing.

### 2.3 Genomic DNA extraction and whole genome sequencing

In order to decipher the genetic structure features of *mcr* genes in these *Salmonella* isolates. We first extracted their genomic DNA using FastPure Bacteria DNA Isolation Mini Kit (Vazyme™, China) according to the instructions of the manufacturer. The purity and concentration of genomic DNA were evaluated using the Titertek-Berthold Colibri (Berthold™, Germany) and the Qubit^R^ Fluorometer (Thermo Fisher™, US) respectively. Then, extracted genomic DNA was sequenced using both short-read and long-read sequencing methods. Short-read DNA sequencing was conducted on the BGISEQ-50 platform with the SE50 strategy. The long-read sequencing was performed on ONT (Oxford Nanopore Technologies) MinION platform using the SQK-LSK109 library preparation kit in R9.4 flow cells.

### 2.4 Genome assembly and genomic feature analysis

The complete genomic sequences of *mcr*-positive *Salmonella* isolates were obtained using Unicycler (Wick et al., 2017) with hybrid assemble strategy based on short- and long-read data. An online fully-automated service, RAST was used to annotate the complete genomes (https://rast.nmpdr.org/). Antimicrobial resistance genes (ARGs), insertion sequences (ISs) and plasmid replicon genes were detected using abricate tool (https://github.com/tseemann/abricate) based on NCBI AMRFinderPlus, ISFinder and PlasmidFinder databases. The plasmid data were downloaded from NCBI RefSeq database (https://ftp.ncbi.nlm.nih.gov/genomes/refseq/plasmid/). The *Salmonella* serovars were identified using SISTR (Yoshida et al., 2016). The multi-locus sequence type (MLST) was analyzed using an mlst tool (https://github.com/tseemann/mlst). BRIG v.0.95 and Easyfig v.2.2.3 were used to generate plasmid comparison maps (Alikhan et al., 2011; Sullivan et al., 2011).

## 3 Results

### 3.1 Identification of *mcr*-positive *S. enterica* and antimicrobial susceptibility testing

In total, 12 *mcr*-positive isolates (ten *mcr-1* and two *mcr-3*) were detected from 1046 clinical *Salmonella* isolates using PCR. The prevalence and basic genomic features have been identified in our previous study (Li et al., 2022). For the 12 *mcr*-positive *S. enterica*, five isolates each year were detected in 2016 and 2017. The remaining two isolates were found in 2014. No *mcr*-positive isolates were detected in 2015. Antimicrobial susceptibility testing showed that the 12 *mcr*-positive *S. enterica* isolates were all resistant to colistin but sensitive to meropenem. In addition, the majority of them were resistant to ampicillin, tetracycline, aztreonam and kanamycin, whereas they were almost all sensitive to ciprofloxacin and enrofloxacin (**Table 1**). The majority of 12 *mcr*-positive *S. enterica* isolates showed multidrug resistance against three or more types of antimicrobials, designated as MDR isolates. According to the conjugation assay, the colistin resistance phenotype of the 12 *S. enterica* isolates could be transferred into *E. coli* C600. Antimicrobial susceptibility testing showed that all transconjugants were resistant to colistin. In addition, majority of transconjugants displayed resistance to other antimicrobials (**Table 1**). This indicated that the resistance genes in donor *S. enterica* were probable carried by conjugative MDR plasmids.

**Table 1:** The basic information and MICs (mg/L) of *mcr*-positive isolates and their transconjugants in this study.

Isolates	Gene	Serovar	STs^a^	Antimicrobials^b^							
				KAN	CIP	MEM	AMP	CL	ENR	TET	CFF
SC2014107	*mcr-1*	Orion	684	8	≤0.25	≤0.25	8	8	≤0.25	4	≤0.25
SC2014238	*mcr-1*	Meleagridis	463	4	≤0.25	≤0.25	>128	4	≤0.25	8	>128
SC2016025	*mcr-1*	I 4,[5],12:i:-	34	128	4	≤0.25	>128	8	2	128	>128
SC2016042	*mcr-1*	I 4,[5],12:i:-	34	8	≤0.25	≤0.25	>128	8	≤0.25	128	8
SC2016090	*mcr-1*	I 4,[5],12:i:-	34	8	≤0.25	≤0.25	>128	4	2	64	>128
SC2016091	*mcr-3*	I 4,[5],12:i:-	34	8	≤0.25	≤0.25	>128	8	≤0.25	128	>128
SC2016290	*mcr-3*	I 4,[5],12:i:-	34	4	≤0.25	≤0.25	>128	8	≤0.25	32	>128
SC2017030	*mcr-1*	I 4,[5],12:i:-	34	>128	≤0.25	≤0.25	>128	8	≤0.25	64	>128
SC2017057	*mcr-1*	I 4,[5],12:i:-	34	>128	≤0.25	≤0.25	>128	1	≤0.25	16	128
SC2017100	*mcr-1*	I 4,[5],12:i:-	34	2	0.5	≤0.25	>128	4	≤0.25	32	>128
SC2017167	*mcr-1*	I 4,[5],12:i:-	34	>128	≤0.25	≤0.25	>128	4	≤0.25	64	>128
SC2017297	*mcr-1*	I 4,[5],12:i:-	34	>128	≤0.25	≤0.25	>128	8	1	32	>128
cSC2014107	*mcr-1*	/	/	1	≤0.25	≤0.25	8	2	1	0.5	≤0.25
cSC2014238	*mcr-1*	/	/	1	≤0.25	≤0.25	32	2	0.5	0.5	0.5
cSC2016025	*mcr-1*	/	/	4	0.5	≤0.25	>128	2	1	32	128
cSC2016042	*mcr-1*	/	/	2	≤0.25	≤0.25	32	2	0.5	0.5	0.5
cSC2016090	*mcr-1*	/	/	4	≤0.25	≤0.25	>128	2	1	0.5	16
cSC2016091	*mcr-3*	/	/	1	≤0.25	≤0.25	>128	2	1	≤0.25	>128
cSC2016290	*mcr-3*	/	/	1	≤0.25	≤0.25	>128	2	0.5	4	>128
cSC2017030	*mcr-1*	/	/	>128	≤0.25	≤0.25	>128	2	0.5	32	32
cSC2017057	*mcr-1*	/	/	8	≤0.25	≤0.25	>128	2	0.5	32	64
cSC2017100	*mcr-1*	/	/	2	≤0.25	≤0.25	>128	2	0.5	0.5	16
cSC2017167	*mcr-1*	/	/	>128	≤0.25	≤0.25	>128	2	0.5	0.5	16
cSC2017297	*mcr-1*	/	/	>128	≤0.25	≤0.25	>128	1	0.5	16	64

a STs indicate sequence types.

b KAN, kanamycin; CIP, ciprofloxacin; MEM, meropenem; AMP, ampicillin; CL, colistin; ENR, enrofloxacin; TET, tetracycline; CFF, ceftiofur.

### 3.2 Genetic context of *mcr-1* and *mcr-3*

**Figure 1:**
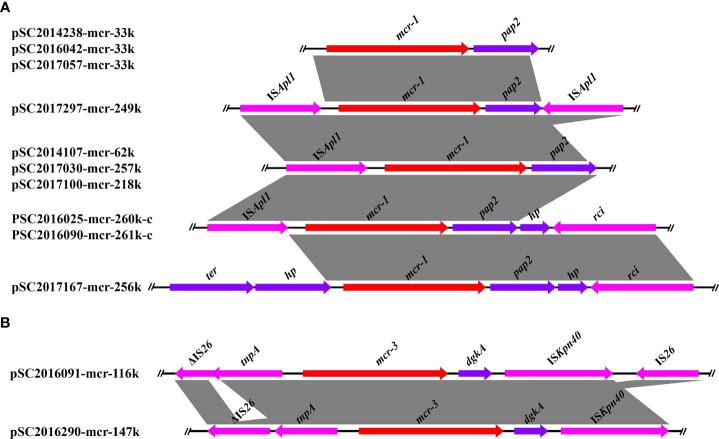
Linear comparison of the core genetic contexts of *mcr* genes investigated in this study. **(A)** The genetic context of *mcr-1* in plasmids and chromosomal plasmids. PSC2016025-mcr-260k-c and PSC2016090-mcr-261k-c were two chromosomally integrated plasmids. **(B)** The genetic structure of *mcr-3* in plasmids pSC2016091-mcr-116k and pSC2016290-mcr-147.

The genetic contexts of *mcr-1* in the ten *mcr-1* positive *Salmonella* isolates were classified as five types (**Figure 1**). The genetic structures of *mcr-1* in IncX4 plasmids were identical. No mobile elements were detected around the *mcr-1* gene in IncX4 plasmids. In IncHI2 plasmids, four different *mcr-1* genetic structures were found. Apart from plasmid pSC2017167-mcr-256k, we found an IS*Apl1* in the upstream of *mcr-1* in the other IncHI2 plasmids. In addition, we detected a reversed IS*Apl1* in the downstream of *mcr-1* in plasmid pSC2017297-mcr-249k. IS*Apl1* was an important element associated with the rapid dissemination of *mcr-1*, which is usually located in both upstream and downstream of *mcr-1* forming an IS*Apl1*-*mcr-1*-*pap2*-IS*Apl1* (Tn*6330*) transposon (Snesrud et al., 2018). Another study demonstrated that *mcr-1* genetic structures with only one IS*Apl1* or with IS*Apl1* absent were formed by deletion of IS*Apl1* from the ancestral Tn*6330* (Snesrud et al., 2018). We detected truncated Tn*6330* in many *mcr-1*-bearing plasmids, suggesting that the genetic structures of *mcr-1* in these plasmids have evolved for a long time.

The genetic structures of *mcr-3* in plasmids pSC2016091-mcr-116k and pSC2016290-mcr-147k were similar and shared a common core structure *tnpA*-*mcr-3*-*dgkA*-IS*Kpn40* (**Figure 1**). In plasmid pSC2016091-mcr-116k, we found a truncated IS*26* present upstream and an intact IS*26* present downstream of *mcr-3*. Only an intact IS*26* present upstream of *mcr-3* was detected in plasmid pSC2016290-mcr-147k. Similar to the mobilization of *mcr-1* medicated by Tn*6330*, the mobility of *mcr-3* is usually associated with a composite transposon structure IS*Kpn40*-*mcr-3.1*-*dgkA*-IS*Kpn40* (He et al., 2020). Apart from IS*Kpn40*, the highly mobilized IS*26* around *mcr-3* deserves our attention, as it may facilitate the horizontal translocation of *mcr-3*.

### 3.3 Distribution of *mcr*-bearing plasmids in *S. enterica* isolates

**Figure 2:**
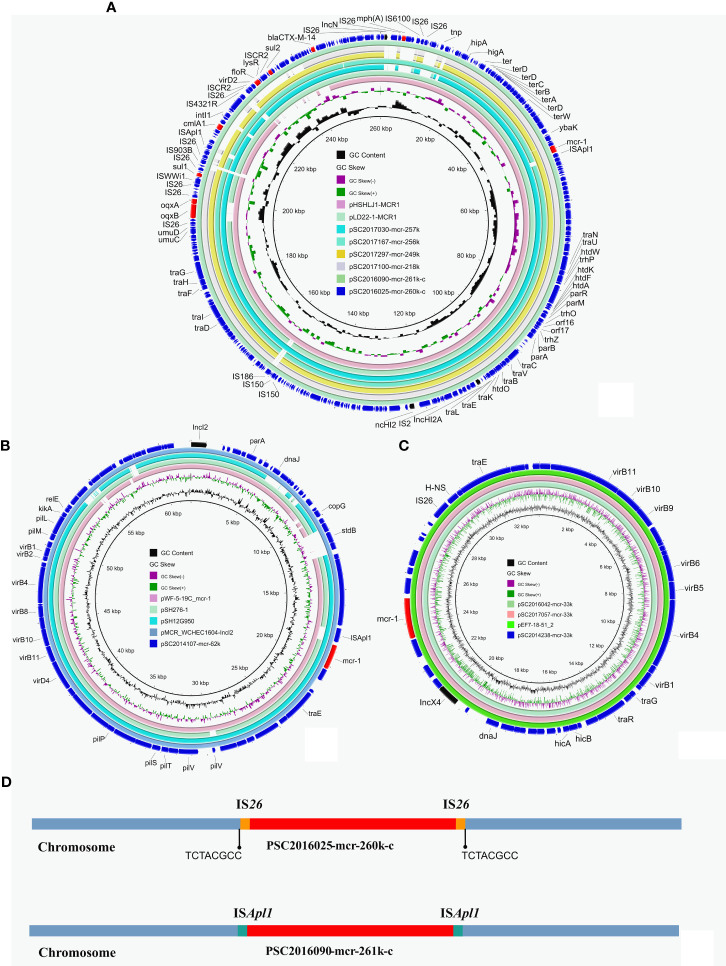
Structure analysis of *mcr-1*-bearing plasmids in *Salmonella* isolates. **(A)** Comparative analysis of *mcr-1*-carrying IncHI2 plasmids and chromosomally integrated plasmids in this study with *other* similar plasmids including pLD22-1-MCR1 (CP047877.1) and pHSHLJ1-MCR1 (KX856066.1). **(B)** Four identical *mcr-1*-bearing IncX4 plasmids including three identified in this study and plasmid pEF7-18-51_2 (CP063489.1) from NCBI database. **(C)** Circular comparative analysis of pSC2014107-mcr-62k with four closely related plasmids including pMCR_WCHEC1604-IncI2 (KY829117.1), pSH12G950 (MH522410.1), pSH276-1 (MG299140.1) and pWF-5-19C_mcr-1 (KX505142.1). Resistance genes and plasmid replicon genes in these plasmids were highlighted in red arrows and black arrows respectively. **(D)** The integration sites of chromosomal plasmids PSC2016025-mcr-260k-c and PSC2016090-mcr-261k-c.

Long-read nanopore sequencing and short-read Illumina sequencing were performed on the 12 *S. enterica* isolates. The complete chromosomes and plasmids of these isolates were obtained using a hybrid assembly strategy and single-read analysis, as described in our previous study (Li et al., 2018). According to the assembly results, we found two chromosomally integrated IncHI2 plasmids from isolates SC2016025 and SC2016090 as a similar phenomenon previously reported (**Figures 2A, D**) (Chang et al., 2022). Similar *mcr-1*-bearing IncHI2/HI2A plasmids were found in other *mcr-1* positive isolates from our investigation including SC2017030, SC2017100, SC2017297 and SC2017167. Moreover, these *mcr-1*-bearing IncHI2/HI2A plasmids were also found in NCBI nr database by BLASTn analysis (**Figure 2A**). Almost identical *mcr-1* bearing plasmids were simultaneously detected in different genus, different geographical locations, different sources of isolates, implying that such plasmids play a crucial role in the horizontal transmission of *mcr-1*.

Comparative analysis found that there are some structure differences in these *mcr-1*-bearing IncHI2/HI2A plasmids from different isolates. A replicon gene of IncN plasmids was absent in plasmids pSC2017100-mcr-218k, pSC2017297-mcr-249k and pSC2017030-mcr-251k. In plasmid pSC2017100-mcr-218k, a region encoding multiple resistance genes containing *cmlA1*, *floR* and *sul2* was lost (**Figure 2A**). The evolution of this type of plasmids in different isolates may result in their structural changing. IncHI2/HI2A plasmids with *mcr-1* are commonly detected in *E. coli* (Lu et al., 2020). This type of *mcr-1* positive plasmid has recently been found frequently in *Salmonella* (Vazquez et al., 2021), indicating that the *mcr-1* gene has the ability to transfer to other species of bacteria with their assistance. We should pay more attention to monitor the spread of such *mcr-1*-bearing plasmids.

We identified three *mcr-1*-bearing IncX4 plasmids in isolates SC2014238, SC2016042 and SC2017057. The three IncX4 plasmids shared 100% identity and 100% coverage with each other. In addition, there are many IncX4 plasmids from other genus of bacteria that are identical to the three plasmids (**Figure 2B**). Like IncHI2/HI2A plasmids, IncX4 plasmids were also a common plasmid type carrying *mcr-1*, because they are self-transferable with high conjugation frequencies (Sun et al., 2017). Apart from IncHI2/HI2A and IncX4 plasmids, an IncI2 plasmid harboring *mcr-1* with 62kb in length was identified in isolate SC2014107 (**Figure 2C**). The IncI2 plasmids have recently been noticed due to they are usually associated with the transmission of ESBLs genes and *mcr-1* (Wong et al., 2015). A previous investigation showed that IncI2 plasmids were the most common type of *mcr-1*-carrying plasmids (Ricker et al., 2022). In addition, we found many *mcr-1*-bearing IncI2 plasmids from different bacteria in nr database (**Figure 2C**), indicating that the IncI2 plasmids were broad-host-range plasmids.

**Figure 3:**
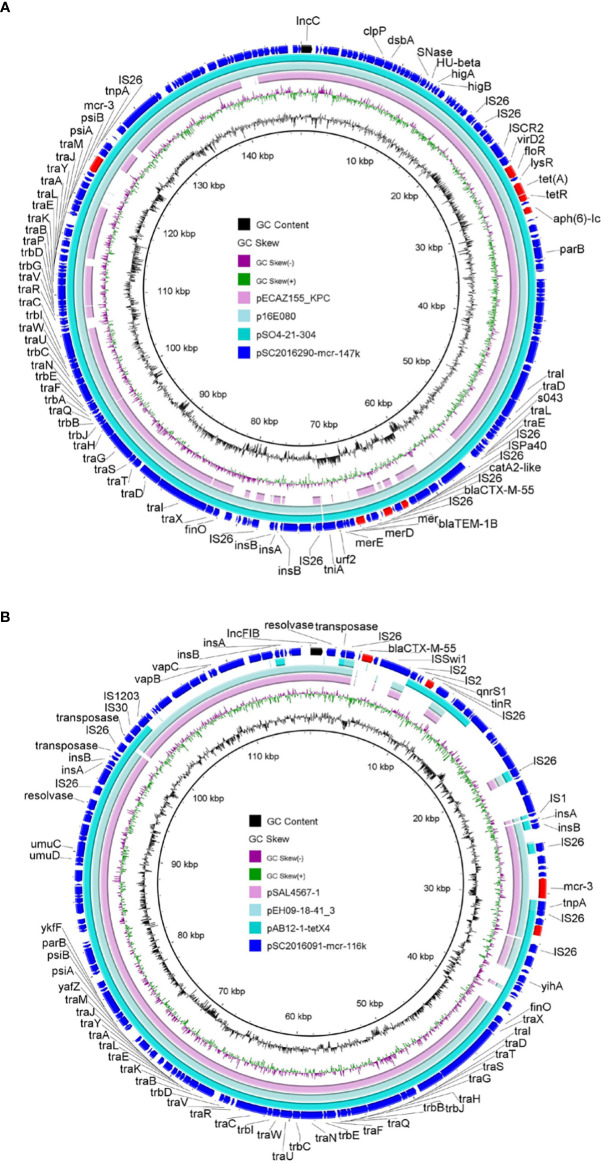
Structure analysis of *mcr-3*-bearing plasmids. **(A)** Comparative analysis of plasmid pSC2016290-mcr-147k with plasmids pSO4-21-304 (AP014634.1), p16E080 (MN647788.1) and pECAZ155_KPC (CP019001.1). **(B)** Structure analysis of plasmid pSC2016091-mcr-116k with other similar plasmids including pAB12-1-tetX4 (MZ054177.1), pEH09-18-41_3 (CP063506.1) and pSAL4567-1 (AP023307.1).

Apart from *mcr-1*, another *mcr* variant *mcr-3* was detected in isolates SC2016090 and SC2016091. Whole genome analysis of the two isolates found that the *mcr-3* gene was located on plasmids. In isolate SC2016090, *mcr-3* was carried by an IncC plasmid pSC2016290-mcr-147k with a size 147kb. Besides, the plasmid also carried *bla*_CTX-M-55_, *bla*_TEM-1B_, *catA2*, *aph(6)-Id*, *tet*(A) and *floR*. We noticed that a few of such *mcr-3*-bearing plasmids have been detected in other *Salmonella* isolates (**Figure 3A**). Of note, the plasmid pSC2016290-mcr-147k showed a partial backbone similar to an *E. coli* plasmid pECAZ155_KPC carrying the carbapenemase gene *bla*_KPC-2_. This phenomenon implied that the plasmid pSC2016290-mcr-147k could integrate into other plasmids, causing the *mcr-3* gene to spread to other bacteria. Another *mcr-3*-bearing plasmid found in isolate SC2016091 was an IncFIB type plasmid pSC2019091-mcr3-116k with 116kb. Coincidently, the plasmid also carried ESBL gene *bla*_CTX-M-55_ (**Figure 3B**). Furthermore, the IncFIB plasmids belonged to a type of broad host plasmids, and there are numerous plasmids from various bacteria sharing a backbone comparable to the IncFIB plasmid.

### 3.4 Prevalence characteristics and structures of *mcr*-positive plasmids

In order to further investigate the prevalence and plasmid backbone structures of *mcr*-positive plasmids, all plasmid data in the RefSeq database were downloaded and utilized for comparative analysis of plasmid backbone structures. Through BLASTn analysis, a total of 136 complete plasmids encoding *mcr* were identified among over twenty-seven thousand plasmids in database. The plasmid replicon types of plasmids were further determined using the plasmidfinder database. The major of *mcr-1* variants identified in both the database and this study was found in plasmids of replicon types IncX4 (34/122), IncI2 (49/122), and IncHI2/HI2A (31/122), and all plasmids of those types carried only *mcr-1*, suggesting those plasmids has a strong correlation with *mcr-1* horizontal transfer. Additionally, eleven plasmids encoding *mcr-3* identified in the database and this study, belonging to four different replicon types, including IncFII (5/11), IncC (4/11), IncI1 (1/11), IncFII/FIB (1/11). Furthermore, the plasmids carrying *mcr-8*, with replicon types IncFII (1/4), IncFII/FIA (2/4) and IncFII/FIB (1/4), and eight additional plasmids carrying *mcr-10* was determined to be of replicon type IncFII/FIB (5/8), IncFII (1/8), IncFII/FIA (1/8) and IncFII (1/8).

The main hosts of *mcr*-positive plasmids in the RefSeq database were identified as *Salmonella enterica* (18/136) and *Escherichia coli* (96/136).

**Figure 4:**
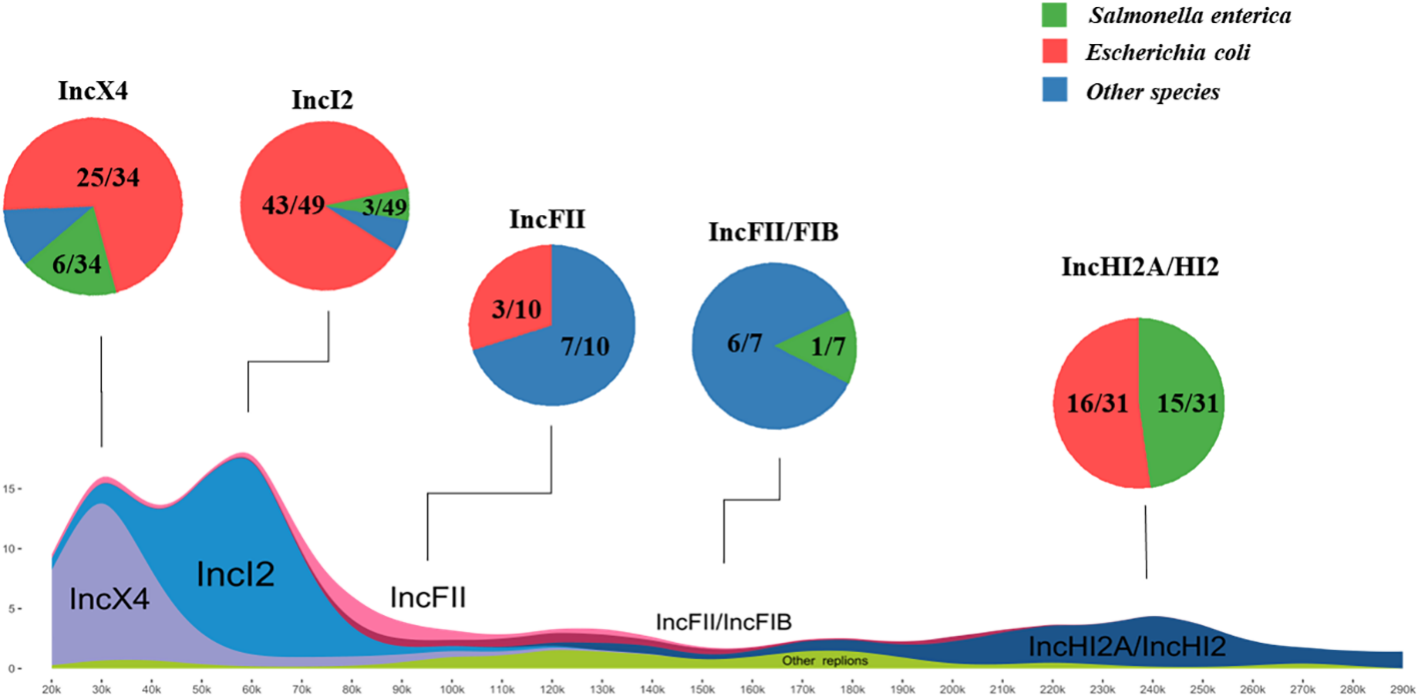
Host distribution and structural characteristics of *mcr*-positive plasmids The investigation of *mcr*-plasmids from RefSeq database *via* BLASTn comparison. A total of 136 complete plasmids encoding *mcr* were identified. *Salmonella enterica* and *Escherichia coli* are the Major hosts of positive plasmids. IncX4 and IncI2 plasmids as most abundant (approx. 30kb or 60kb), followed by IncHI2A/HI2 (220kb-270kb). The frequent detection of *mcr* in various plasmid backbones and bacterial isolates highlights the widespread distribution and prevalence of this resistance mechanism.

The plasmids from both the database and this study were merged for further investigations. Among these plasmids, those belonging to replicon types IncX4 and IncI2 were most abundant, often in the size range of approximately 30kb and 60kb, respectively, followed by IncHI2A/HI2 type plasmids mainly distributed between 220kb to 270kb in length (**Figure 4**). Alignment of plasmid backbone structures revealed that, aside from IncFII/FIB replicon type *mcr*-positive plasmids, those with the same plasmid replicon types displayed similar backbone structures (**Supplement Figure 1**). These plasmids with similar structures were found in different host bacteria especially *Escherichia coli*, indicating the horizontal transmission of *mcr*-positive plasmids carrying different *mcr* variants among different hosts.

### 3.5 Genetic characteristics of multidrug resistant chromosomally integrated plasmid

**Figure 5:**
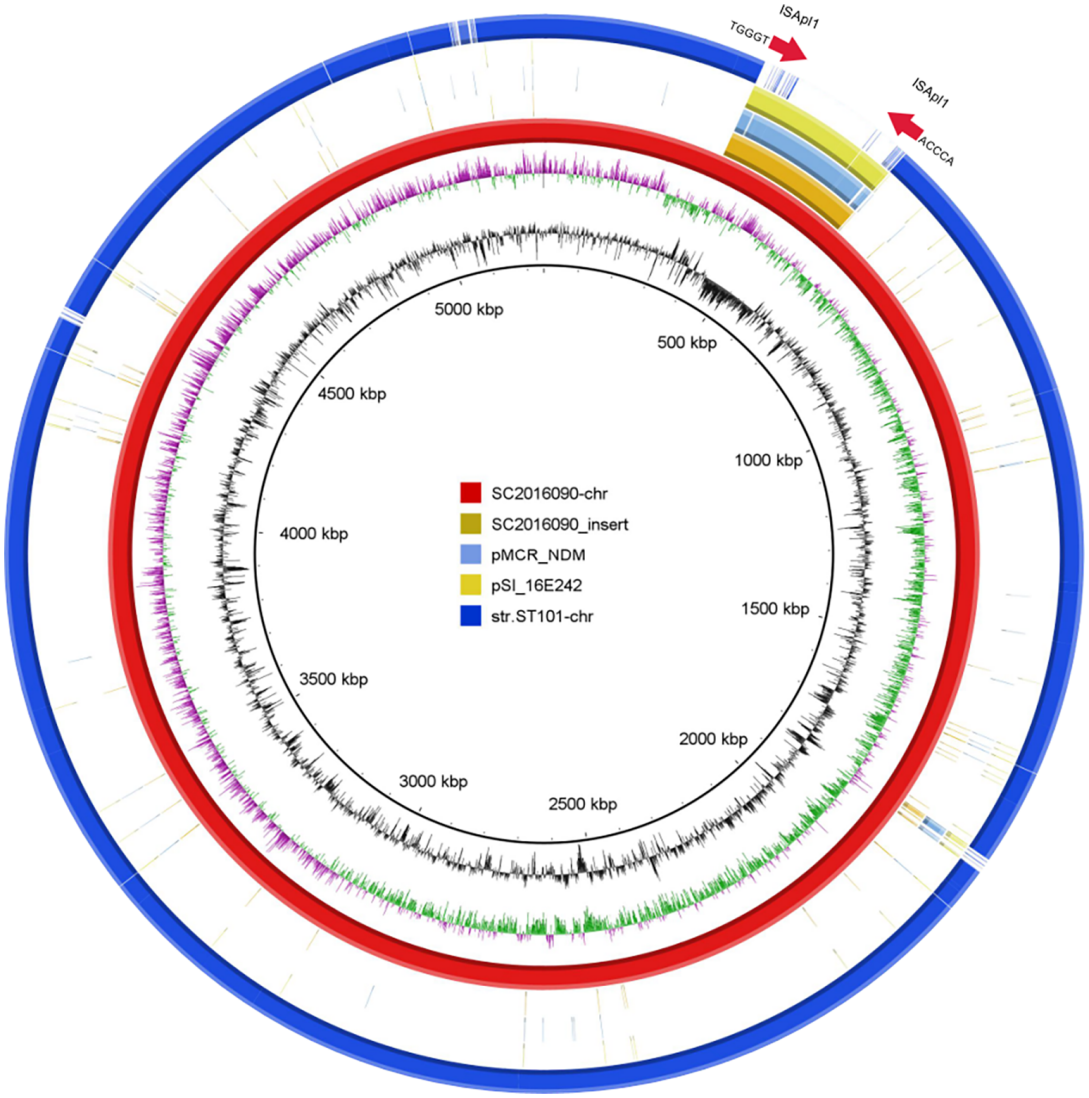
The presence of the IS*Apl1* gene and structure analysis of multidrug-resistant chromosomally integrated plasmid. BLASTn revealed high homology (93% coverage and 99.97% identity) between SC2016090 and *Salmonella enterica* strain ST101 chromosomes (CP050731.1). The extra fragment showed 99% identity with plasmids pMCR_NDM (CP049111.1) and pSI_16E242 (ON960347.1), with respective coverage of 95% and 99%. The presence of the IS*Apl1* transposase and reverse repeating base sequence was detected at both ends of the fragment.

To investigate the formation mechanism of the integrated plasmid, we employed third-generation DNA sequencing to obtain accurate genome structures of strain SC2016090. The chromosome of SC2016090 was 5,247,576-bp in length, exhibited a GC content of 51.9% and comprised 4,927 predicted coding sequences (CDSs). Comparative analysis using BLASTn revealed that the chromosome of SC2016090 showed remarkable similarity (93% coverage, 99.97% identity) to the chromosome of *Salmonella enterica* strain ST101 (CP050731.1), isolated from a diarrheal patient in Shanghai. However, the SC2016090 chromosome contained an extra 261,335 bp DNA fragment with 46.8% GC content (**Figure 5**) that was not present in the ST101 chromosome.

Further investigation revealed that this extra fragment encompassed 249 CDSs and displayed 99% identity with plasmids pMCR_NDM (CP049111.1) and pSI_16E242 (ON960347.1), with coverage of 95% and 99%, respectively. Notably, within the extra DNA fragment of strain SC2016090, several others resistance genes, including *bla*_CTX-M_ *, sul3, aadA2*, and *fosA* were detected, IncHI2 replicon was also coding in the extra DNA fragment. Furthermore, a comprehensive analysis of the extra DNA fragment unveiled the presence of numerous mosaic genetic elements, including the clustered Tn*26*. Interestingly, we found the CDS for a D-serine transporter (DsdX, QIU08742.1) was truncated by insertion of the extra DNA fragment. Additionally, the presence of the IS*Apl1* in both areas of the fragment’s ends and the reverse repeating base sequence located on both sides of the extra plasmid segment, suggested the involvement of IS*Apl1* in the insertion of the integrated plasmid (**Figure 5**).

## 4 Discussion

Although colistin was not the first-line antibiotic for the treatment of *Salmonella* infections, emergence of colistin resistant *Salmonella* in clinical posed a great threaten to human health. To date, many studies have proved that colistin resistance genes *mcr* have spread in *Salmonella* of animal and human sources (Lu et al., 2019; Yang et al., 2022). This alarmed that colistin resistant *Salmonella* should not be neglected. Plasmids play an important role in the dissemination of *mcr* in many bacteria, including *Salmonella*. Understanding the *mcr*-bearing plasmids characteristics of *Salmonella* is important to prevent the diffusion of *mcr* in *Salmonella*. In this study, we comprehensively investigated the characteristics of *mcr-*bearing plasmids in 1046 clinical *S. enterica* isolates collected in Sichuan province from 2014 to 2017. We found that *mcr-1* was predominated *mcr* variant in clinical *Salmonella*. Three different types of *mcr-1*-bearing plasmids (IncHI2/IncHI2A, IncX4 and IncI2) were found in the 10 *mcr-1* positive *Salmonella* isolates. Of note, the three types of plasmids were commonly detected in *E. coli* (Sun et al., 2017; Lu et al., 2020), implying that *mcr-1* in *Salmonella* was probably came from *E. coli*. Apart from *mcr-1*, we also detected two *mcr-3* positive *Salmonella* isolates. The *mcr-3* gene in the two *Salmonella* isolates were carried by two different types of plasmids (IncC and IncFIB). It is worth noting that the *mcr-3*-bearing plasmids detected in *Salmonella* were also broad-host plasmids. In addition, both *mcr-1*- and *mcr-3*-harboring plasmids in our study could be transferred into *E. coli* by conjugation assay, indicating that *mcr* genes in clinical *Salmonella* isolates might spread into other bacteria.

According to our investigation, we found majority of *mcr*-positive *Salmonella* isolates belonged to *S.* 4 [5],,12:i:-. Previous study showed that MDR phenotype of *S.* 4,[5],12:i:- was determined by IncHI2 plasmids they carried (Mu et al., 2022). We also found a high detection rate of IncHI2 plasmids in *mcr-1* positive clinical *S.* 4,[5],12:i:-. This alerted us to pay more attention to monitor the spread of IncHI plasmids in different ecological niche.

In summary, the prevalence of *mcr* genes was low in clinical *Salmonella* in Sichuan, China. The *mcr-1* was more prevalence than *mcr-3*. The propagation of *mcr* genes in *Salmonella* was mainly mediated by plasmids. The *mcr*-bearing plasmids detected in clinical *Salmonella* could transfer among different Enterobacteriaceae bacteria. Continuous surveillance of *mcr*-bearing *Salmonella* in different settings as the One Health approach should be performed to curb the potential risk caused by MDR *Salmonella*.

## Data availability statement

The datasets presented in this study can be found in online repositories. The names of the repository/repositories and accession number(s) can be found in the article/**Supplementary Material**.

## Ethics statement

The manuscript presents research on animals that do not require ethical approval for their study.

## Author contributions

XY, RL, and ZW contributed to conception and design of the study. XS, LZ, JM performed the experiments and organized the data. KP and WH conducted the sequencing and bioinformatics analysis. WH and GL did data curation and visualization. XS and KP wrote the first draft of the manuscript. RL and XY reviewed and edited the manuscript. All authors contributed to the article and approved the submitted version.
