# Supplementary material for: The characteristics of mcr-bearing plasmids in clinical Salmonella enterica in Sichuan, China, 2014 to 2017
Source: Front Cell Infect Microbiol. 2023 Aug 29;13:1240580. doi: 10.3389/fcimb.2023.1240580 (PMC10495832; doi:10.3389/fcimb.2023.1240580)
Supplement: Supplementary file 1 [file DataSheet_1.docx]

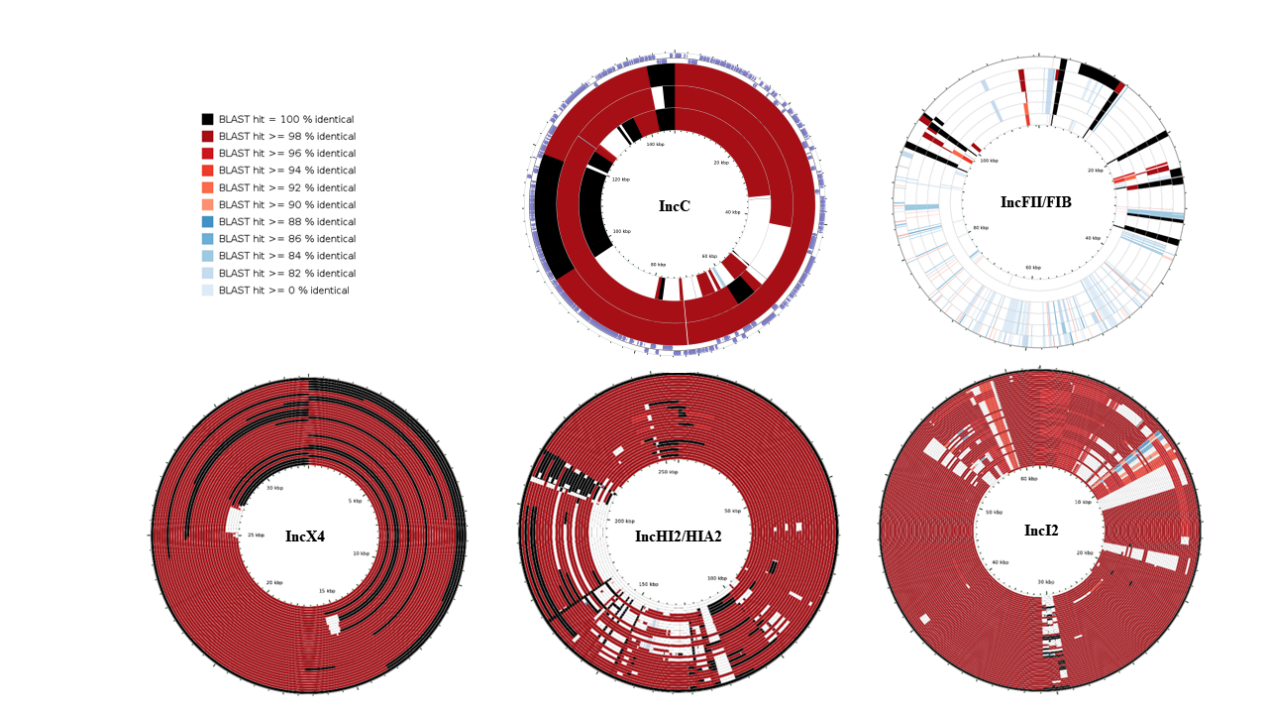


**Supplement Figure 1：Comparison of different types of *mcr*-positive plasmids**

The alignment of plasmid backbone structures demonstrated that *mcr*-positive plasmids with replicon types other than IncFII/FIB exhibited similar backbone structures, and structurally analogous plasmids were identified across various host bacterial species.
